# Trend judgment as a perceptual building block of graphicacy and mathematics, across age, education, and culture

**DOI:** 10.1038/s41598-023-37172-3

**Published:** 2023-06-24

**Authors:** Lorenzo Ciccione, Mathias Sablé-Meyer, Esther Boissin, Mathilde Josserand, Cassandra Potier-Watkins, Serge Caparos, Stanislas Dehaene

**Affiliations:** 1 Cognitive Neuroimaging Unit, CEA, INSERM, NeuroSpin Center, Université Paris-Saclay, 91191 Gif-sur-Yvette, France; 2 Collège de France, Université Paris Sciences Lettres (PSL), 75005 Paris, France; 3 LaPsyDÉ, CNRS, Université Paris Cité, 75005 Paris, France; 4 Laboratoire Dynamique Du Langage, UMR 5596, Université Lumière Lyon 2, 69363 Lyon, France; 5 DysCo Lab, Department of Psychology, Université Paris 8, 93526 Saint-Denis, France; 6 Human Sciences Section, Institut Universitaire de France, 75005 Paris, France

**Keywords:** Psychology, Human behaviour

## Abstract

Data plots are widely used in science, journalism and politics, since they efficiently allow to depict a large amount of information. Graphicacy, the ability to understand graphs, has thus become a fundamental cultural skill comparable to literacy or numeracy. Here, we introduce a measure of intuitive graphicacy that assesses the perceptual ability to detect a trend in noisy scatterplots (“does this graph go up or down?”). In 3943 educated participants, responses vary as a sigmoid function of the *t*-value that a statistician would compute to detect a significant trend. We find a minimum level of core intuitive graphicacy even in unschooled participants living in remote Namibian villages (N = 87) and 6-year-old 1st-graders who never read a graph (N = 27). The sigmoid slope that we propose as a proxy of intuitive graphicacy increases with education and tightly correlates with statistical and mathematical knowledge, showing that experience contributes to refining graphical intuitions. Our tool, publicly available online, allows to quickly evaluate and formally quantify a perceptual building block of graphicacy.

## Introduction

Humans often exhibit a surprising intuitive grasp of the core concepts of mathematics, physics or statistics. These intuitive abilities, which emerge in the absence of formal education, are likely to rely on a system of core implicit knowledge about the fundamental properties of the environment in which humans evolved^1^. A solid body of research shows that humans can accurately and quickly grasp the approximate numerosity of sets of objects^2^, and perform approximate calculations even in the absence of formal mathematical education^3^; indeed, even populations without a rich lexicon for number and arithmetic seem to possess a strong number sense^4,5^. Euclidean and non-Euclidian geometrical intuitions of space are present in remote Amazon populations without access to formal education^6,7^. In concrete settings, humans also excel in intuitive physics: their misconceptions about the behavior of moving objects^8^ disappear when questions are framed in familiar and real-life contexts^9^. Humans are also exceptionally skilled at making intuitive statistical estimations for a variety of activities^10^ and these abilities emerge early in development^11^. Furthermore, quantitative assessments of intuitive mathematics and physics are predictive of the subsequent development of higher-level cognitive abilities^12–16^, indicating a strong connection between initial “core” intuitions and the subsequent mastery of related formal concepts (with the former acting as perceptual and cognitive precursors for the latter).

Whether such intuitions extend to graphical representations is still an open question, however. While data graphs are increasingly abundant in our cultural environment, no quantitative assessment of human statistical intuitions of graphical trends has been proposed. Here, we show how the recent development of graph-based psychophysics tasks offers a tool to quantify intuitive graphicacy at its perceptual level, in a way that (unlike most previous studies in the data visualization literature) does not request any numerical answer nor any explicit knowledge of graphs and charts.

**Figure 1:**
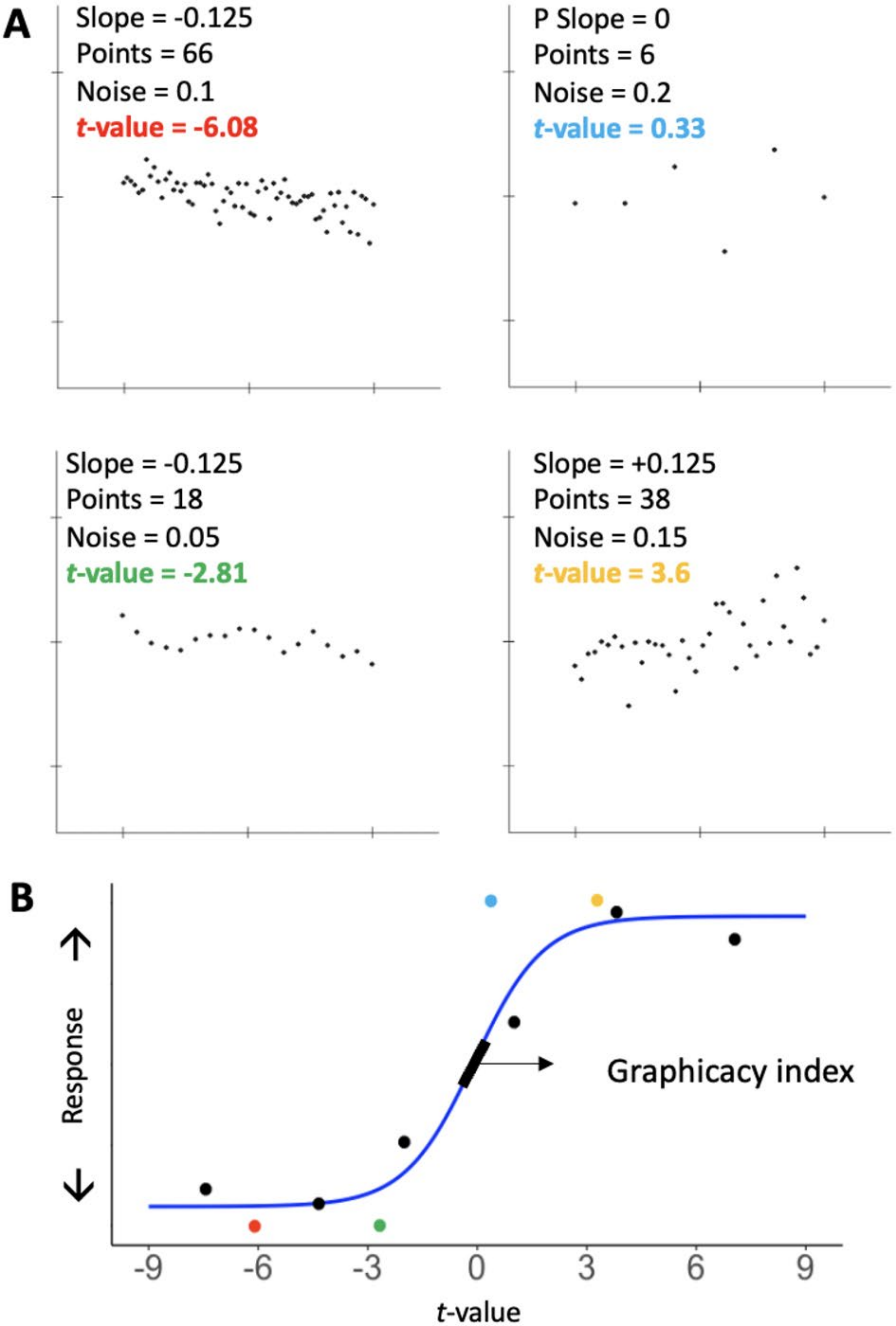
A simple test of intuitive graphicacy. (**A**) Four examples of stimuli shown to one participant in the trend judgment task, where the participant was asked if the graph was increasing or decreasing. The actual stimuli were white dots on a black background. Each scatterplot was created according to the combinations of different parameters: slope of the generative function, number of points, and noise level. Each thus had a certain* t*-value, corresponding to the *t* statistic used to calculate the significance of the trend in the dataset. (**B**) Responses given by a representative participant are plotted as a function of the *t*-value of each scatterplot. Color dots show the data for the four example trials in panel A. For visualization purposes, the black dots show averages over bins of* t*-values. We fitted the data with a psychometric function (blue curve). The slope of the sigmoid, indicated by a black bar, evaluates the subject’s sensitivity in the trend judgment task, and was used as a “graphicacy index”, a proxy of the participant’s intuitive graphicacy.

We previously showed that, when facing a graph such as a noisy scatterplot (Fig. 1A), human adults can detect whether the graph depicts an increasing or decreasing trend, across large variations in the number of data points, the noise level, or the slope of the graph. Their performance in such a simple perceptual task is predicted by the *t*-value that a statistician would calculate to determine the significance of the trend in the data^17^. In other words, the percentage of “increasing” responses is a sigmoid function of the *t*-value of the scatterplot. While our previous work relied on group-level analyses, here we reasoned that, as in any two-alternative forced-choice task, the slope of the psychometric function (Fig. 1B) should provide a measure of individual participants’ sensitivity to detect variations in the stimulus: the steeper the function, the higher the participant’s precision.

In the present work, we show that this measure can be reliably used to assess human intuitions of noisy graphs. We used it to investigate the distribution of trend judgment skills across people of different ages, education levels and cultures, suggesting it may serve to quantify a key perceptual building block of graphicacy and mathematical knowledge.

First, we tested trend judgments in a large-scale online sample of educated adults from all over the world, from which we obtained information about several demographical aspects (including age and gender), together with self-reports of mathematical and statistical understanding. Testing intuitive graphicacy on such a large and diverse population offered insights into the predictors of these skills; also, it provided a large-scale replication and extension of psychophysical results previously obtained with a reduced sample in a controlled laboratory environment^17^, thus contributing to the growing but still scarce body of research on psychophysical measurements outside the lab^18–21^.

Second, we explored whether this ability to detect trends in noisy graphs emerges as a result of graph exposure at school and/or college, or whether some perceptual premises of graphicacy are available even in the absence of formal education. If trend judgments are grounded on basic perceptual abilities, then even people with no exposure to math or graphs should be able to make those judgments. To investigate this, we tested Himba participants, a Namibian people with no or little formal education, who are not exposed to any form of graphical representations. This sample of participants allowed us to test for the generalizability of such perceptual skills in non-western and unindustrialized societies, as previously been done for other intuitive skills^1^, including the perception of number^3^, time^22^, and geometry^6,7,23^.

In addition, we tested intuitive graphicacy in French 6-year-old 1st-graders who never encountered any graphical representation in their school curriculum. We thus asked whether the ability to compute intuitive visual statistics from graphs arises early on in development, as should be the case if it relies on core skills of human perception, similar to number sense or shape detection. The cultural recycling hypothesis^24^ postulates that the evolutionary ancient cognitive functions of numerosity perception and shape recognition serve as a foundation for the corresponding cultural skills (respectively, arithmetic and reading). We similarly postulate that humans’ ability to read and interpret complex graphs might be based on fundamental cognitive and perceptual functions available early on in development, irrespective of formal education: the recognition of the orientation and the medial axis of objects (their “skeleton”^25–27^). In essence, a scatterplot, although made of many data points, would be processed as an oriented object, and its perception would recycle the evolutionary ancient ability to estimate in which direction the main axis of this object points to. Indeed, one aspect of our previous results fits with this hypothesis: the slope that participants attribute to a graph does not correspond to the classical ordinary least squares (OLS) value, but to an estimate of the graph’s principal axis, also known as Deming regression^17^. Showing that the same graphical intuition is already available to 1st-graders, prior to any formal education in mathematical graphs, would constitute further evidence for its core nature.

## Results

### Performance in the trend judgment task is predicted by the *t*-value of the scatterplot

**Figure 2:**
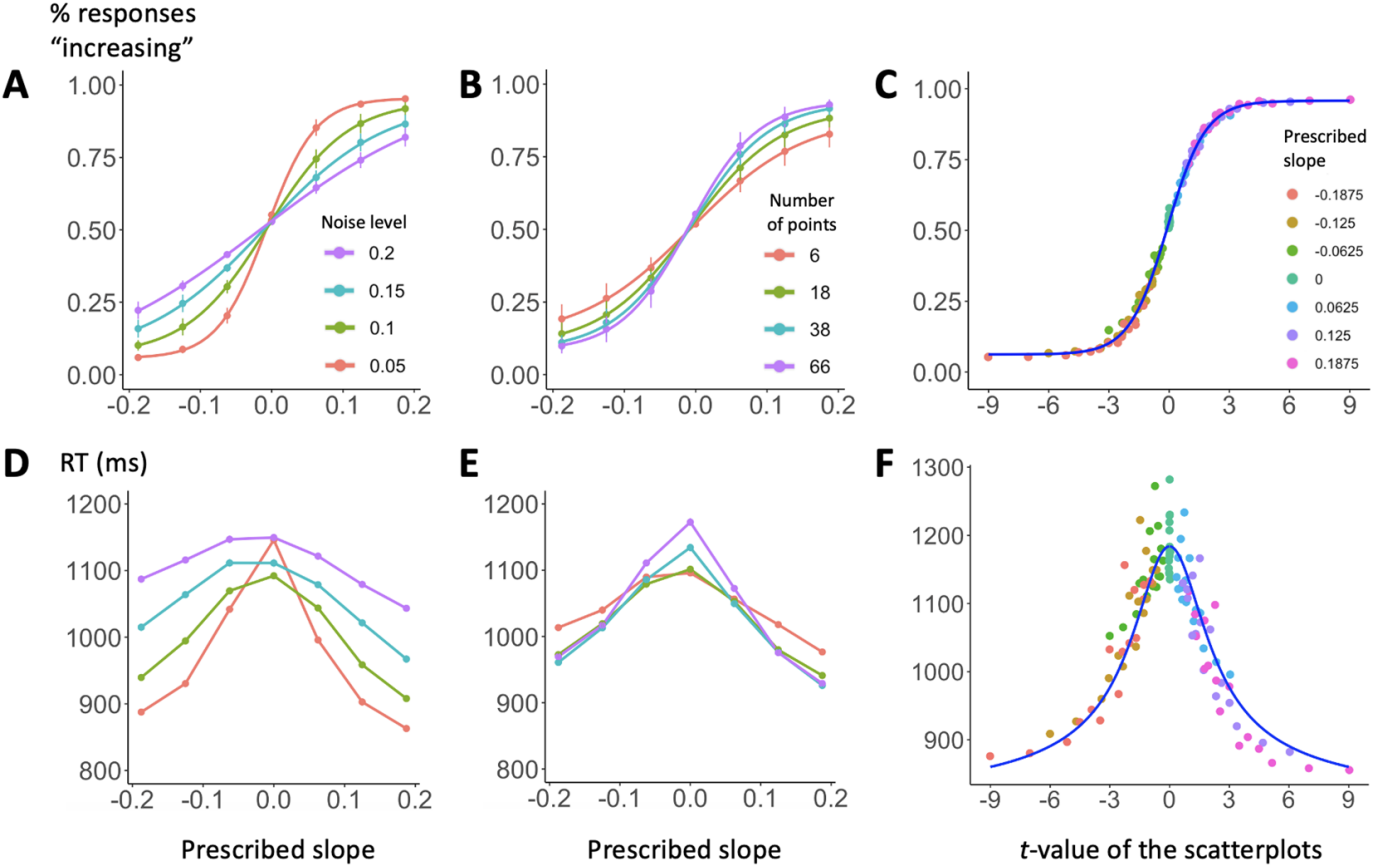
Psychophysics of graph perception (N = 3943). The top row shows the percentage of responses “increasing” as a function of prescribed slope and noise level (**A**), prescribed slope and number of points (**B**), and *t*-value associated to the Pearson correlation coefficient of the scatterplot (**C**). Panel C shows that participants’ performance can be subsumed by the *t*-value. (**D**–**F**) Equivalent plots for response times (RT) as a function of prescribed slope and noise level in plot (**D**), prescribed slope and number of points in plot (**E**), prescribed slope and* t*-value in plot (**F**). The blue line in plot (**F**) shows the prediction of a simple accumulation of evidence model (Gold and Shadlen, 2002).

As described in the “Methods” section, 3943 adults participated in an on-line experiment where they had to decide whether scatterplots, with variable number of data points, slopes and noise levels, showed an ascending or descending trend. We first plotted the percentage of “increasing” responses as a function of the prescribed slope (i.e., the steepness of the scatterplot), the noise level and the number of dots (Fig. 2). We replicated results from previous research conducted on a small sample of subjects in a laboratory context^17^, finding that the proportion of “increasing” responses was affected by all the above parameters (Fig. 2A and B). In an ANOVA on the proportion of “increasing” responses as a function of the prescribed slope, the noise level and the number of points, all factors had a significant main effect, and the prescribed slope significantly interacted with both the noise and the number of points (all *p* < 0.001). These findings confirm what is clearly visible in Fig. 2A, B: the smaller the slope of the graph, the higher the influence of the noise level and the number of points on the trend judgment task. With more points shown and with less noisy scatterplots, participants were more accurate at detecting the trend in the graph. No interaction effect was found between the noise and the number of points (F[8.5, 19,667.8] = 0.81, *p* = 0.6), suggesting that the two factors independently affected human trend judgments.

All of these effects, however, were subsumed by an effect of the *t*-value associated to the Pearson coefficient of correlation (Fig. 2C), which varies positively and linearly with the prescribed slope, positively with the number of points (as the square root of n − 2) and inversely with the noise level. Accordingly, we computed a multiple logistic regression on “increasing” responses as a function of the *t*-value, the number of points and the noise level (averaged within each of the 112 experimental conditions, both across stimuli and across all subjects), and we found that only the *t*-value was a significant predictor of participants’ responses (β_t-value_ = 0.77, *p* < 0.0001; β_number of points_ = 0.002, *p* = 0.83; β_noise_ = − 0.48, *p* = 0.91). When we included the subjects as random factors in the regression analysis, all predictors became significant (*p* < 0.0001) but crucially, when examining the odds ratios (which indicate the effect size of predictors in logistic regressions), only the* t*-value exhibited a very large odds ratio of 16.05. In contrast, both the noise and the number of points showed negligible effects, with odds ratios of 0.97 and 1.06, respectively (note: in logistic regressions, odds ratios of 1 indicate absence of effect).

The classical t test formula ($t= \frac{\widehat{\mathrm{\alpha }}}{{s}_{\widehat{\mathrm{\alpha }}}}=\sqrt{n-2}\frac{r }{\sqrt{1-{r}^{2}}}$ with $r=\frac{cov\left(x,y\right)}{{\sigma }_{x} {\sigma }_{y}})$ implies that t grows as the square root of the number of points minus 2. In anticipation of this rule, we used four values for the number of points that were linearly spaced after this square-root transformation (n = 6, 18, 38, 66) and, indeed, we found that performance varied according to this parameter (Fig. 2B).

As far as response times for correct answers are concerned (Fig. 2D and E), we submitted them to separate linear regressions as a function of the three main experimental factors (the absolute value of the main slope, the noise level and the number of points) and with the subjects as random factors. We found that, while both the prescribed slope and the noise level significantly predicted response times (β_slope_ = − 946.5, *p* < 0.0001; β_noise_ = 920.3, *p* < 0.0001; i.e., participants were slower for noisier and shallower plots), this was not the case for the number of points (β_number of points_ = 0.06, *p* > 0.05), thus suggesting a parallel processing of all items in the set. A simple model of noisy evidence accumulation^17,28^ (see those references for modeling details) correctly fitted the average response times on all trials (blue line in Fig. 2F), based solely on the percentage of responses given by the subjects.

### The trend judgement task is a reliable measure that varies widely across individuals

**Figure 3:**
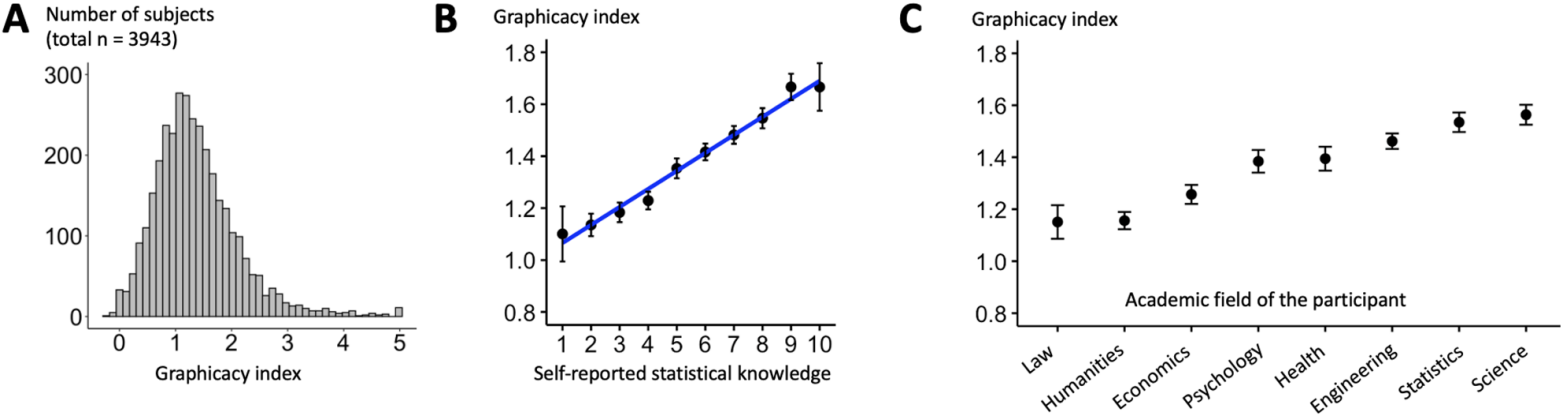
Inter-individual variability in intuitive graphicacy. (**A**) Distribution of the graphicacy index across participants. (**B**) Intuitive graphicacy increases as a function of participants’ self-reported statistical knowledge, collected before the experimental task was introduced. (**C**) Intuitive graphicacy in participants that obtained at least a bachelor degree varies as a function of the academic field in which they graduated (F(7,2891) = 15.57, *p* < .001; note that data were ordered according to each group’s mean graphicacy index).

We modeled participants’ responses as a sigmoid function of the *t*-value of each stimulus they saw (Fig. 1B). We postulated that the steepness of this function reflects their intuition of trends in noisy graphs (as a perceptual precursor of graphicacy), and we therefore called the slope of the sigmoid for a given participant their “graphicacy index”. Figure 3A shows the distribution of this index across the large sample we collected online (median value = 1.26). For the vast majority of participants (98.2%), the regression was significant and with a positive index, thus providing a reliable estimate and confirming that they were not responding randomly. However, intuitive graphicacy (measured as the slope of the psychometric function, as just described) varied considerably across individuals, with 95% of the distribution falling between 0.20 and 3.18.

To evaluate the stability of this index during the course of an experimental run and test for training effects, we computed the index separately on the first 50 trials and on the following 50 trials (for the 3419 participants that performed only one experimental run). Although the increase was significant (Wilcoxon signed rank test, *p* < 0.0001), it was small, passing from a median of 1.28 to 1.35. Most crucially, there was a significant correlation between the two values (r(3417) = 0.38, *p* < 0.0001), thus showing a stability of inter-individual variations in intuitive graphicacy. To further evaluate whether the graphicacy index remained stable over time, we computed the slope of the orthogonal (Deming) linear regression between those two measurements and we found that the regression slope was close to one (1.02; 95% confidence interval = [0.94, 1.1]), thus suggesting that, on average, the graphicacy index was quite stable and reproducible within an individual. This conclusion was further supported by analyzing the data from participants who completed more than one block of trials (n = 387). The correlation between their graphicacy index in the first experimental run and in the second one was high (r(385) = 0.49, *p* < 0.0001) and the slope of the orthogonal linear regression between them was again close to one (1.16; 95% confidence interval = [0.95, 1.36]).

Overall, these results suggest that our measure of intuitive graphicacy is stable, at least in the absence of extensive training, and can be reasonably estimated in a 6-min on-line test. It is likely that, should one require a more stable individual measure, a longer testing session would provide an even more reliable graphicacy index.

### Intuitive graphicacy correlates with statistical knowledge and academic field

We then tested whether trend judgment skills correlated with participants’ self-evaluation of their skills (assessed before the main psychophysical test). There were highly significant correlations between participants’ graphicacy index and their self-reported statistical knowledge (Fig. 3B; r = 0.21, df = 3092, *p* < 0.0001) and mathematical knowledge (r = 0.22, df = 2028, *p* < 0.0001). How specific was this correlation? A large majority of subjects (N = 2030) also answered a self-evaluation question on their first language skills, always using a scale from 1 to 10. We performed a multiple linear regression on the graphicacy index as a function of statistical knowledge and language skills, finding that the former was a significant predictor (β = 0.07, *p* < 0.0001) but the latter was not (β = − 0.006, *p* = 0.55). This finding suggests that participants’ graphicacy was not simply predicted by general personal skills or self-confidence.

Figure 3C shows how the mean graphicacy index varied as a function of the academic field in which graduate participants obtained their title (F(7,2891) = 15.57, *p* < 0.001): it was considerably higher for graduates in engineering, statistics and science (n = 1576, mean = 1.51) than for graduates in other disciplines (n = 1323, mean = 1.27; t(2894.4) = 8.92, *p* < 0.0001). In graduate subjects, the graphicacy index also significantly correlated with their reported average grade in mathematics (r = 0.24, df = 3028, *p* < 0.0001).

### Correlations between the graphicacy index and other factors

The graphicacy index also correlated with participants’ self-reported ability to understand scatterplots (r = 0.23, df = 3225, *p* < 0.0001) and their familiarity with scatterplots (r = 0.23, df = 3217, *p* < 0.0001). The graphicacy index was also an inverted U-shaped function of age: it increased until the age of ~ 35 and then decreased (R^2^ with a quadratic trend = 0.38, *p* < 0.0001). It also significantly increased with higher levels of formal education (r = 0.14, df = 3417, *p* < 0.0001). Concerning gender, no significant difference of the graphicacy index was observed between women and non-binary participants (women = 1.28, non-binary = 1.23, t(97.8) = 0.73, *p* = 0.47) but we found a significant, although small, advantage in favor of men over women (men = 1.54, t(2145.9) = 9.44, *p* < 0.0001) and over non-binary participants (t(109.8) = 4.28, *p* < 0.0001). Although these results are in agreement with previous research suggesting better spatial abilities in men than in women (for a review^29^), they are inconclusive given that the present sample was self-recruited and not representative. Also, such difference was not found in the Himba (t(83.4) = 0.6, *p* = 0.55), nor in children (t(24.4) = 0.3, *p* = 0.77), thus suggesting that a higher ability to perform the task in online male participants could arise due to socio-cultural factors. We found no significant difference in graphicacy index between participants responding on a touchscreen and on a computer (t(2510.9) = − 0.9, *p* = 0.37), nor between participants asked to respond with their left versus their right hand/finger for scatterplots going up (t(3410.6) = 0.11, *p* = 0.91).

### Himba performance is predicted by the *t*-value of the scatterplot, independently of age and education level

**Figure 4:**
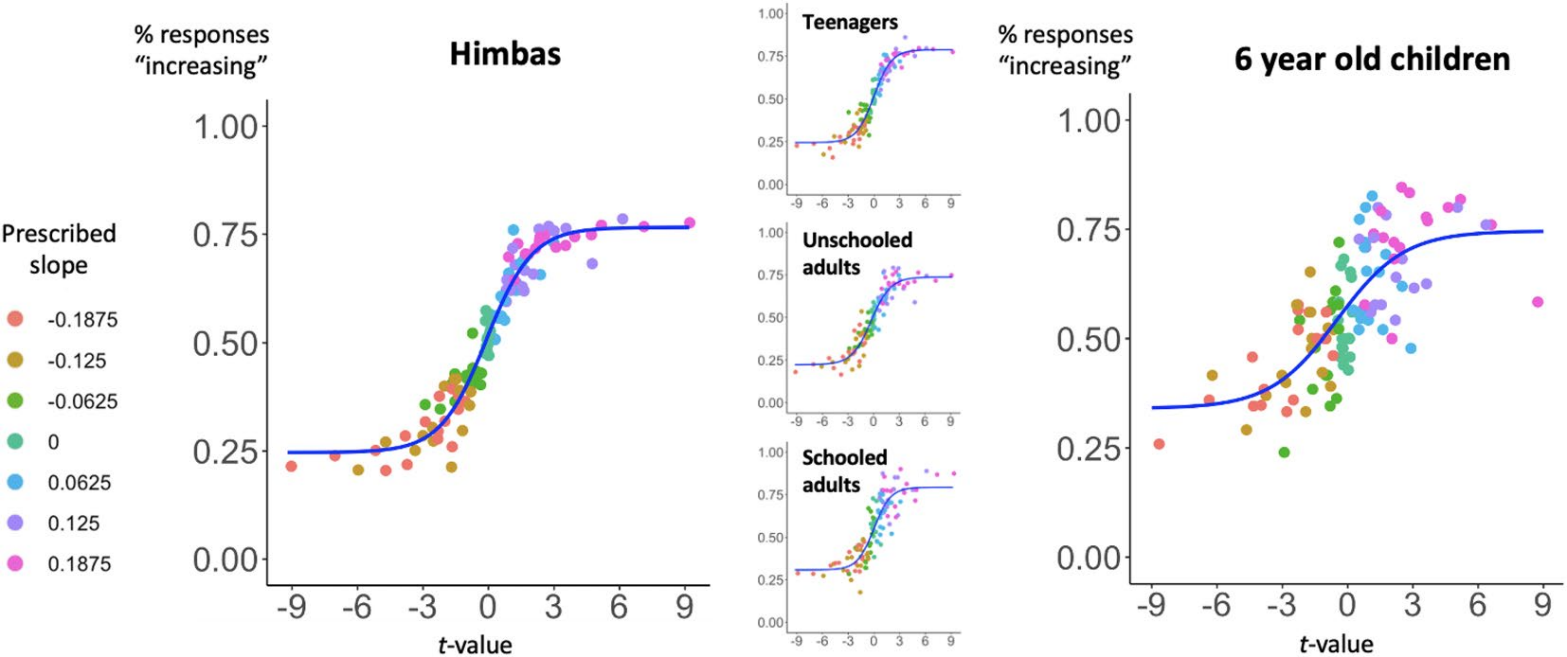
Precursors of graphicacy in the absence of formal education. The percentage of responses “increasing” is well predicted by the *t*-value associated with the graph Pearson correlation coefficient *r*, both in 6-years-old children (right, N = 27) and in the Himba participants (left, N = 87), an ethnic group from Namibia with reduced access to formal education. Insets show the effect separately as a function of schooling and age for the Himba.

We used the same exact test in 87 members of the Himba culture from Namibia, with reduced access to formal education. Figure 4 (left plot) shows that Himba performance in trend judgment was again well predicted by the *t*-value of the scatterplot.

We again computed a multiple logistic regression of responses “increasing” as a function of the *t*-value, the number of points and the noise level, and we found that, again, the *t*-value was the only significant predictor of participants’ responses (β = 0.27, *p* < 0.01), while the noise (β = 0.37, *p* = 0.92) and the number of points (β = 0.001, *p* = 0.86) were not. The same findings held when we separated our data in three separate groups (Fig. 4, middle plots): teenagers (i.e., participants younger than 18 years old, N = 36), unschooled adults (i.e., participants who did not receive any formal education, N = 39), and partially schooled adults (i.e., participants who attended mobile schools during at least 1 year, N = 12). For all these subgroups, responses were significantly predicted by the *t*-value of the stimulus (all β with *p* < 0.01). The median graphicacy index for the Himba was 0.32, i.e., on the very low end of the distribution of educated subjects (see Fig. 3A), but significantly positive (t(86) = 11.53, *p* < 0.0001), even in unschooled adults (t(38) = 7.29, *p* < 0.0001). Thus, formal education is not needed for participants to perform the task according to an intuitive and approximate statistical t test for linear regression.

### 6-years-old children’s performance is also predicted by the *t*-value of the scatterplot

The results were also replicated in a group of 27 6-years-old 1st-grade children (Fig. 4, right plot). Although children’s average responses were noisier and never reached perfect performance (as is clear from the boundaries of the sigmoid function in Fig. 4, right plot), their responses were again significantly predicted by the* t*-value of the scatterplot, which sufficed to account for children’s performance: it was a significant predictor of their responses (β = 0.17, *p* < 0.05), whereas the noise (β = 1.97, *p* = 0.58) and the number of points (β = − 0.0003, *p* = 0.98) were not.

For each child, we computed the graphicacy index and correlated it with the two measures described in the Methods’ section: groupitizing advantage (an implicit measure of their arithmetic abilities) and reading speed (number of correctly read words in 1 min). Both correlations were significant (respectively: r = 0.51, df = 25, *p* < 0.01, and r = 0.46, df = 25, *p* < 0.05). It is worth noting that groupitizing and reading speed were also highly correlated (r = 0.7, df = 25, *p* < 0.0001). The median graphicacy index for children was of 0.12, therefore significantly lower than the index for the Himba (0.32; t(47.7) = 2.76, *p* < 0.01), but still significantly above zero (t(26) = 3.9, *p* < 0.001).

## Discussion

In our study, we measured the human ability to perform a trend judgment task on a noisy graph (i.e., “Does this graph go up or down?”). Analyzing the responses of 3943 participants who performed the task online on computers or tactile devices, we found that their accuracy was affected by all three manipulated factors, namely the steepness of the graph, its noise level and the number of points. In terms of response times, there was a significant effect of steepness and noise but not of the number of points. This finding suggests that participants treated the scatterplots as an ensemble, without serially processing each item: in fact, if that was the case, we should have observed an increment in response times proportional to the number of points in the dataset. In this respect, as recently proposed^30^, fast intuitive statistical judgments on graphs with Gaussian noise seem to operate similarly to ensemble perception, the human core ability to rapidly extract the “average” of visually displayed items, without focusing on each particular element in the set^31,32^. There are key differences, however (as revealed in the presence of outliers in the graph^33^), the most important one being that trend judgments cannot rely on an estimation of the “average element” (as it would happen when participants are asked to estimate the average color, size or orientation of a set of objects), but focus on the overall shape and trend of the cloud of data points.

Crucially, participants’ responses were significantly predicted by the *t*-value associated to the Pearson coefficient of correlation of the graph, showing that human trend judgments approach those of an optimal statistical model^34^. While some data show that people do not include sample size in their variability estimations^35^, thus suggesting that they wrongly assume that a small sample is always representative of the entire population^36,37^, our studies demonstrate that, for datasets represented in a bivariate scatterplot format, people correctly incorporate both variability and sample size in their trend judgments. In other words, at least at a perceptual level, humans are not naïve in their statistical estimates, but seem to take into account all the parameters of the dataset.

It is worth noting, tangentially, that the psychophysical results from our online participants replicated the findings from a trend judgment task performed in a laboratory context^17^. This piece of evidence is important both empirically and methodologically. It confirms that psychophysical studies do not need to be confined to a controlled laboratory environment and can be successfully performed online, as it has been recently done in the field of data visualization^38,39^. This clearly reduces research times and costs, especially when participants, such as in the present online study, were included on a purely voluntary basis and with no other reward than personal enjoyment.

The first drive of our study was to introduce a quantitative measure of trend judgment, a perceptual skill that may serve as a foundation for higher-level graph understanding. Using a psychophysical approach, we operationalized our quantitative measure of graphicacy as the slope of the psychometric function linking subjects’ “increasing” responses to the graph* t*-value^40^ and we found that this measure varied in the general population and reflected the participants’ self-evaluation of statistical and mathematical knowledge (but not, crucially, their self-evaluation of first language skills) and, for those who studied some mathematics at university, with their grades in math exams. Although such self-evaluations may be criticized, much research shows that they are a good proxy of objective achievement measured through standardized tests^41–44^. Our results suggest that the development of intuitive graphicacy correlates with math expertise, similarly to the positive correlation between mathematical education and the accuracy of numerosity perception^13,45^. As with numeracy, the causal link and direction of this relationship remains to be determined and might well be bidirectional. Whether a better grasp of numerical concepts strengthens graph-based statistical judgments, or vice versa, thus remains an open question that could be better addressed in the future through a finer assessment of participants’ numerical skills and their longitudinal follow-up. This is particularly necessary in the case of children: in our sample, we found a strong correlation between intuitive graphicacy and both arithmetic and reading performance, which does not allow to conclude for a specific correlation of the performance in the trend judgment task with numerical cognition; in fact, based solely on our results, one might argue that trend judgments in children correlate with measures of intelligence and/or academic knowledge. Interestingly, however, a relation between complex graph understanding and numerical cognition does indeed seem to exist^46^. While a link between intuitive graphicacy and more complex statistical graph understanding remains to be shown, we believe that our trend judgment task could be an adequate assessment tool for the former, being simple and fast to perform. Similarly to a previous numerosity perception test^20^, we have made the test publicly available online (https://neurospin-data.cea.fr/exp/graphicacy-index/), so that it can be freely run by all researchers interested in investigating the correlation between their participants’ trend judgment skills and other abilities. Future research could also assess causality by examining whether training on this task would generalize to higher-level graph understanding, in the same way that training the intuitive number sense has been suggested to increase arithmetic skills^47^, but see^48^.

It is important to emphasize, once more, that our trend judgment task is assessing only one building block of graph perception, namely the perceptual ability to detect linear trends in bivariate noisy scatterplots: in fact, the understanding of cultural artifacts usually rely on an entire set of perceptual and cognitive building blocks. To make a parallel, the precision of the approximate number system correlates with the understanding of more complex mathematics^49^, but it is far from being the only determinant of numeracy and math understanding^50^. Analogously, graphicacy goes well beyond statistical intuitions over noisy scatterplots. Beyond the perceptual stage, understanding a graph requires being able to grasp the relationship between number and position on the x and y axis^51,52^, to extract meaning from the observed relationship among those two quantities and, possibly, to make quantitative inferences and qualitative decisions based on that derived meaning. We believe that the performance in our trend judgment task provides, at least, one formal assessment of the perceptual stage of graphicacy. Future studies should also explore the larger realm of perceptual and cognitive skills that together form the precursors of graph comprehension, including spatial abilities, numerical intuitions, number-space mappings, attentional skills, and see if and how they correlate with each other and collectively predict performance on higher-level graph comprehension tasks.

The second motivation of our study was to investigate whether intuitive graphicacy was only available to individuals previously exposed to graphical representations, or whether it could be found in the absence of any such exposure. 10-years-old children were previously shown to discriminate graphical representations based on the location and size of the datapoints^53^, but no study to date tested whether they can perform statistical judgments on them. We show here that French 6-years-old children (unexposed to graphical representations) and uneducated Himba (living in an unindustrialized society in Northern Namibia where graphs are absent), like educated adults, base their intuitive decisions on the *t*-value of the scatterplot (although with an overall poorer performance, which could be due in part to a lack of familiarity with digital devices^54^). Thus, statistical intuitions of graphical linear trends seem to be universally available and to emerge early on in development, irrespectively of previous exposure to graphical representations. The present finding echoes previous data supporting the existence of a universal understanding of quantities^2,3^, geometrical shapes^23^, probabilities^11^, biology^55^, and human psychology^56^. However, we also found that the graphicacy index was much lower in Himba and children than in educated adults. Taken together, our results strongly suggest that intuitive graphicacy does not solely mature as a function of age, but can be refined by education and/or exposure to statistics and graphical representations. Recent evidence^57^ suggests that the precision of numerical estimations increases with education through an improved ability to focus on relevant information in the task and to discard non-numerical features. Future studies may investigate whether a progressive refinement of the filtering of irrelevant information (e.g., noisy or outlier datapoints) is also responsible for the relationship between the graphicacy index and education.

As suggested in the introduction, we propose that the universality of statistical intuitions over noisy scatterplots reflects a cultural recycling of pre-existing perceptual abilities for object orientation. According to this hypothesis, noisy graphs would be treated as objects, and detecting an increasing or decreasing trend would simply mean detecting the orientation of this object relative to the horizontal axis. Humans are in fact particularly sensitive to changes in stimulus orientation^58–60^. Furthermore, they seem to rely on the object’s principal axis when asked to detect its orientation^61,62^. This observation agrees with our earlier finding that humans extract the principal axis of a graph when invited to fit a trend line over a noisy scatterplot^17^: the line that participants fit does not minimize the sum of the squares of the vertical deviations to the data points (as in ordinary least squares), but the sum of the squared perpendicular distances to the data (which is the very definition of the principal axis). In the future, it would be interesting to examine whether other psychophysical properties are shared between graph perception and object orientation tasks, for instance by presenting objects varying in their level of “noise”. It is not trivial, however, to define what noise means for solid objects: for instance, orientation discrimination of tilted gratings is largely unaffected by variations of contrast^63^ but is altered by spatial filters applied on those gratings^64^.

It should also be noted that graphs are a very special type of object to which humans are variably exposed during their life. Experience with these strange “objects” might explain why we found a strong correlation between trend judgment performance and mathematical knowledge. Future research should probe if a higher familiarity with math refines solely the ability to deal with noisy graphs or, alternatively, if it leads to broader improvements in orientation perception for any type of objects. This issue is similar in spirit to the impact of literacy on mirror invariance, which primarily affects the perception of letters such as b and d, as well as letter strings, but also extends to object and face perception^65^.

In summary, by investigating the premises of human intuitive graph perception, our study lays the foundations for a quantitative assessment of one of the main perceptual building blocks of graphicacy, i.e., trend judgment. This is an essential goal in building effective and early educational interventions that might in turn strengthen the comprehension of the complex graphs that humans are more and more routinely confronted with.

## Methods

### Experimental procedure and participants

#### Online participants

The online test was advertised and shared on social networks, mainly through Twitter. It could be performed either on computers or on tactile devices. The study was approved by the local Ethical Committee (*Comité d’Ethique pour la Recherche de l’Université Paris-Saclay*) under the reference CER-Paris-Saclay-2019-061. Before taking part in the experiment, a written informed consent was obtained from the participants (who declared to be at least 18 years old). All experiments were performed in accordance with the relevant guidelines and regulations. Data collection for the purpose of the study started on January 15th, 2021 and ended on March 15th, 2021, as planned ahead of the experiment. The link to the test was still running after that date, but the data were not included in the current work.

Before taking the test, all participants answered a demographic questionnaire consisting in a series of single-answer questions about: country of origin, age, gender, number of previous participations in the task (if any) and the highest level of education attained. If participants declared to have completed a university degree, they were asked to choose the closest field of the degree within a list, and, for STEM graduates, their average grade in mathematics during their university years. Using a Likert scale (ranging from 1 to 10, with intermediate numbers not shown), all participants had to rate their subjective self-evaluation in the following domains: familiarity with graphs, ability to read scatterplots, knowledge of statistics, current skills in mathematics, and current skills in their first language in terms of spelling, grammar and communication. Once the demographic questionnaire was completed, participants started the experiment. Smartphone and tablet users were asked to rotate their phone horizontally: otherwise, the task would not start; accidentally orientating the phone vertically during the task lead to a pause in the experiment. The instructions and the questionnaire were available in six languages: English, French, Italian, Spanish, Portuguese and Chinese. 3943 subjects participated and completed the online experiment (the ones that did not complete the task were not included in the data analysis). 2409 of them declared being women, 1294 men, 82 non-binary, 20 “other” than the previous ones, and 138 preferred not to answer. The average age was 28.8 ± 9.6 years.

#### Himba

87 Himba participants (39 women and 48 men) were recruited in small villages in the Kunene region, Northern Namibia. Most Himba do not know their age. Participants’ age, 21.1 ± 9.4 years, was evaluated by local research assistants who were bilingual Namibians (in Otjiherero and English). Those assistants also instructed each participant, in their native language (Otjiherero), about how to perform the task on a tablet. Before the experiment, each participant was provided with four examples of stimuli and the expected correct answers (i.e., an arrow going up and an arrow going down): no other information was provided to explain the task. Each participant indicated whether they had received any type of formal schooling. Rudimentary mobile schools (using black board and chalk) exist in the Kunene region, and only 12 participants declared having received at least 1 year of such form of schooling.

#### Children

27 French 1st graders (6 ± 0.6 years; 13 girls) took part in the experiment (the study was approved by the local Ethical Committee under the reference CER-Paris-Saclay-2021-046) and completed the experimental tasks. Each child was accompanied by an experimenter to a silent room and invited to sit on a chair facing a table. Before starting the actual experiment, they performed three short behavioral tests: a 1-min reading task consisting in a series of French words of increasing difficulty; a 1-min counting task on sets of points of increasing numerosity; and a 1-min counting task on those same sets of points, but organized in groups (e.g., 4 groups of 3). The first task provided a number of correctly read items in 1 min, which was used as a proxy of reading abilities. The difference in correctly enumerated items between the second and the third task provided an implicit measure of the mastery of arithmetic operations, because grouped items can be enumerated faster if children know how to perform mental arithmetic (“groupitizing”^66,67^). The main experimental task was performed on a tablet and, immediately before it, each child was provided with four examples of stimuli and their expected correct answers; no other information was provided to explain the task. The sample size of Himba and children participants was based on a previous study that used an identical trend judgment task^17^.

### Experimental task

Each trial consisted in the rapid presentation (100 ms) of a scatterplot (Fig. 1A). Participants performed a trend judgment task: they had to judge, as fast and accurately as possible, whether the scatterplot was increasing or decreasing by pressing one of two separate keys on their computer keyboard or, if they played on a smartphone/tablet, by touching an upwards or a downwards arrow. For the online experiment, the response configuration of the keys and the arrows was randomly determined at the beginning of the experiment for each subject, in order to control for possible preferential response sides; also, each correct response was rewarded with a certain amount of points, inversely proportional to the response time. Such gamification incited participants to be both accurate and fast. To maintain a high level of attention in the task, consecutive correct responses were rewarded with increasingly higher points. Also, a pleasant sound followed each correct trial and an unpleasant sound followed each incorrect trial. For children and Himba participants, a smiling green face or a red unsmiling face was displayed instead of the numerical score. A fixation cross was presented for 1000 ms before the following trial appeared. The experimental session lasted around 6 min. Online and Himba participants had the opportunity to start another run or to stop. Online participants could also check their percentage of correct responses and their ranking relative to all previous participants. For data analysis, we rejected any answer that was given after more than 5 s from stimulus onset (0.75% of trials for online participants; 9.39% for children; 0.91% for the Himba).

It is worth emphasizing how the present task differs from those previously used in the graphicacy literature. Graph intuitions are often assessed by asking participants to rate the subjective correlation strength in a scatterplot^68^, to express its slope ratio as a percentage^69^, or to perform statistical judgments in the context of plausible real-world graphical representations of data^38^. However, correlational judgments are hard to relate to objective statistics^70,71^, they linearly depend on actual correlation measures only for r > 0.95^72^, and they are affected by prior beliefs^73^. Furthermore, these kinds of judgments require a numerical response, refer to explicit mathematical or graphical concepts, and are therefore challenging to perform by unschooled populations such as those that we wanted to test in our study, who never saw a graph in their life. For all these reasons, a non-numerical trend judgment task with no information about the nature of the data seemed more appropriate: it avoided subjective perceptions of correlation or slope and merely asked for a forced-choice binary decision on a noisy stimulus, thus allowing for classical psychophysical analysis.

### Stimuli

The stimulus generation algorithm was identical to the one used in a previous laboratory version of the task^17^. Each scatterplot was the graphical representation of a dataset randomly generated from a linear equation of the form y_i_ = α x_i_ + ε_i_, where α is the prescribed slope and the ε_i_ are random numbers drawn from a normal distribution centered on zero and with standard deviation σ. A total of 112 scatterplots were presented to participants, which were the result of the combinations of 3 orthogonal factors: 7 prescribed slopes (α = − 0.1875, − 0.125, − 0.0625, 0, + 0.0625, + 0.125 or + 0.1875); 4 levels of noise (σ = 0.05, 0.1, 0.15 or 0.2); and 4 numbers of points (n = 6, 18, 38, 66). All coordinates on the x axis were fixed and equally spaced for each level of n. Figure 1A shows four examples of stimuli. For each scatterplot, the *t*-value associated to its Pearson coefficient of correlation was calculated. Figure 1 shows examples of stimuli and responses for one subject. Answers are plotted as a function of the *t*-value of the corresponding trial. For each subject, we fitted a classic psychometric function to the data (shown in blue in Fig. 1A) and we extracted its slope, which provided a measure of precision at performing the trend judgment task. The first 12 trials for each subject were considered as practice trials and thus excluded from the computation of this index. Also, a minority of subjects who participated in the online experiment had a very large sensitivity index, meaning that their performance was close to perfect (in fact, it was better modelled by a step function rather than by a sigmoid one). To avoid excessive variability, sensitivities higher than 5 (0.03% of all participants) were capped at 5.

## Data Availability

The datasets generated and/or analyzed during the current studies are available on the Open Science Framework repository at: https://osf.io/cw6t5/.
